# Alexia Without Agraphia: A Rare Entity

**DOI:** 10.7759/cureus.1304

**Published:** 2017-06-02

**Authors:** Chintan Rupareliya, Syeda Naqvi, Seyedali Hejazi

**Affiliations:** 1 Department of Neurology, University of Missouri, Columbia, Missouri; 2 Jinnah Postgraduate Medical Centre, Jinnah Sindh Medical University (SMC); 3 Neurology, Suny Upstate Medical University, Syracuse, NY

**Keywords:** stroke, occipital lobe, alexia, agraphia, cerebral ischemia, visual word form area, visual changes, hemianopia, disconnection syndrome, posterior cerebral artery

## Abstract

Pure alexia refers to an acquired disorder associated with the damage to medial occipitotemporal gyrus in the dominant hemisphere, which is also known as visual word form area (VWFA). VWFA is involved in rapid word recognition and fluent reading. Alexia without agraphia is a disconnection syndrome that occurs when the splenium is also damaged with the occipital lobe on a dominant side.

We report a case of a 72-year-old right-handed male who presented with alexia without agraphia accompanied by right homonymous hemianopia resulting from acute infarct of the left occipital lobe, the splenium of the corpus callosum and posterior thalamus that probably occurred on the previous day. During the evaluation, he exhibited marked impairment in the ability to read with the vision being grossly normal. Magnetic resonant imaging (MRI) revealed an acute infarct of the left occipital lobe, the splenium of the corpus callosum and posterior thalamus. A computerized tomography angiogram (CTA) revealed left posterior cerebral artery (PCA) territory infarct without any evidence of hemorrhagic conversion.

Infarction of the occipital lobe on the dominant side (left) in a right-handed individual may cause a disruption in the visual word form area and is manifested by an inability to read with no abnormalities in visual acuity.

## Introduction

Alexia without agraphia or pure alexia is an acquired disorder secondary to a defect in the left occipitotemporal region affecting the visual word form area (VWFA). In this condition, most of the patients have right-sided homonymous hemianopia due to the involvement of the occipital lobe [1]. This disconnection syndrome is exceptionally rare among acquired neurological deficits in stroke patients.

Alexia without agraphia is characterized by the loss of reading abilities in a literate person, he/she can name things well but cannot read properly. Hence in pure alexia, the patient has preserved visual recognition and writing skills. Magnetic resonance imaging (MRI) and computerized tomographic angiography (CTA) play an essential role in investigating brain lesions causing pure alexia [2].

## Case presentation

A 72-year-old male with a history of hyperlipidemia and Type 2 diabetes mellitus presented to the emergency department of the Community General Hospital (CGH) with generalized malaise and some difficulty with reading for one week. The malaise was first noted by the patient and he had consulted his primary care provider (PCP). The PCP had ordered his blood test including complete blood count, random blood sugar, basic metabolic panel, and thyroid stimulating hormone. The PCP discharged him after excluding any serious illness. However, the patient continued to feel the generalized malaise and a reading difficulty and thought that he needed glasses.

**Figure 1:**
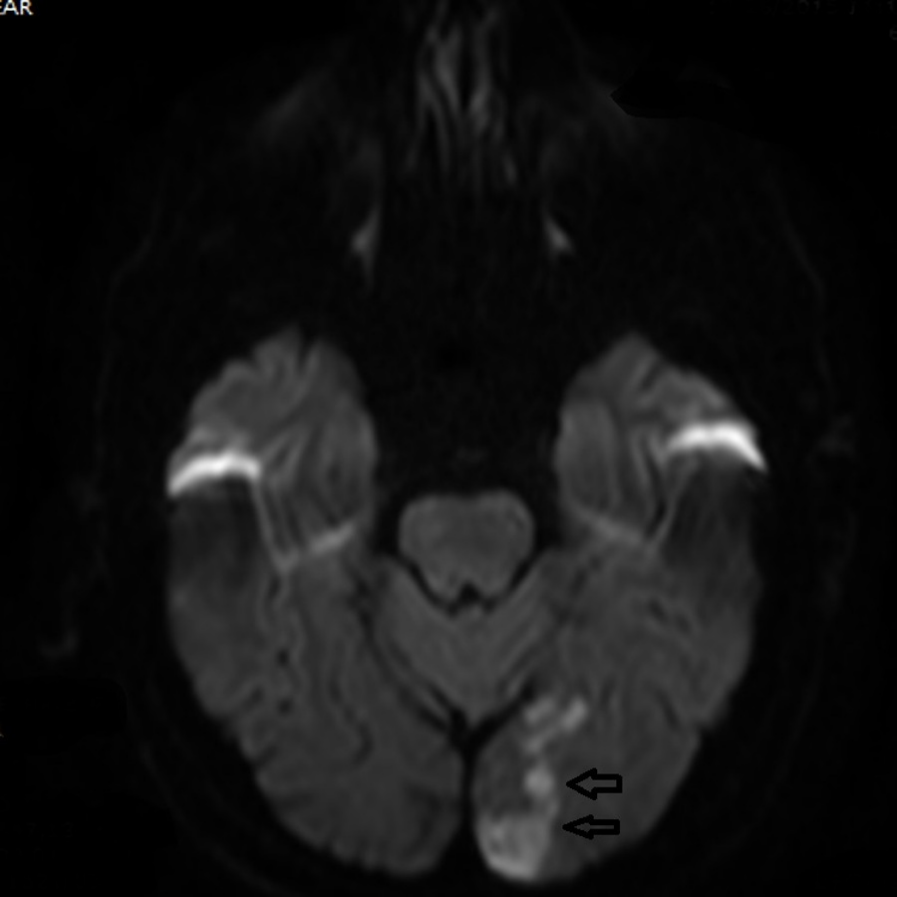
Magnetic Resonance Imaging of Brain Arrows point toward the area of infarct.

Within two weeks, the patient noticed that he was not able to do daily activities as before. His wife noticed that he has some extent of confusion and delayed response. She also noticed that the patient was not as active as before. She convinced the patient to visit the emergency department at CGH where he underwent CTA and MRI of the brain with and without contrast. The MRI in Figure 1 revealed an acute infarction of the left occipital lobe with the involvement of the splenium of the corpus callosum and posterior thalamus.

After these findings, the patient was transferred to Upstate University Hospital for a higher level of care. On the day of admission, he was alert and oriented. The optic discs were normal. He was noted to have right-sided homonymous hemianopia in both eyes. Both the pupils were equal, round, and reactive to light. His extraocular eye movements were full with no nystagmus. He was found to have an inability to read written words and phrases even though his ability to write was found to be intact. The rest of his neurological examination including language, sensory, and motor functions were intact. A magnetic resonance imaging of the brain performed on the prior day had revealed an acute infarction of the left occipital lobe extending into the splenium of the corpus callosum and posterior thalamus. The cerebral angiogram revealed an acute infarct in the territory of the left posterior cerebral artery and an incidental finding of high grade left internal carotid artery stenosis.

He was admitted into neurology inpatient service to find out the cause of the stroke and for treatment by standard stroke protocol. The hospital course was uncomplicated and was not accompanied by any new findings. His symptoms of generalized malaise improved but the right homonymous hemianopia remained unchanged. Meanwhile, all the workup for revealing an etiology of the stroke came negative including transthoracic as well as a transesophageal echocardiogram. The patient was discharged and put on telemetry for continuous monitoring of any paroxysms of atrial fibrillation.

## Discussion

Alexia without agraphia is a clinical disconnection syndrome which was first described by Dejerine in 1892 followed by Geschwind in 1965. These and other authors have mentioned these rare findings where patients are unable to read their own handwritten words. It is usually accompanied by visual field defects like right-sided homonymous hemianopia [1,3]. Alexia without agraphia is a neuro-ophthalmologic condition in which the patient loses the ability to read; however, the ability to write is grossly intact. The rarity of this condition is justified by the fact that it is an indirect involvement of an angular gyrus in the dominant hemisphere which represents an area for word recognition, affecting the reading capacity only contrary to reading and writing ability impairment occurring together [4].

Most cases of alexia without agraphia are caused by left posterior cerebral artery (PCA) occlusion and a resultant infarct of the left visual cortex as well as the splenium of the corpus callosum, which is the case here. Involvement of the splenium disrupts the connection between the intact right visual cortex with the left angular gyrus, which is the cortical center for reading [5-6]. The preserved writing ability is attributed to the intact left angular gyrus, as it is the connection pathway which is destroyed with the splenium infarction and not an angular gyrus itself, which obtains its supply from the middle cerebral artery.

Even though alexia without agraphia is usually produced by an infarction of the left occipital cortex along with the splenium of the corpus callosum, other exceptional causes include acute encephalopathy [7], multiple sclerosis flares with relapsing remitting nature evident by white matter changes in the left visual cortex [8], and glioblastoma [9].

This patient presented with acute onset of alexia without agraphia of nonprogressive nature. The MRI finding at the Community General Hospital (CGH) classically showed infarction of the left occipital lobe with an extension into the splenium of the corpus callosum. Patients who present with an inability to read with preserved visual acuity should be investigated further for a possibility of this rare phenomenon.

Based on the literature review, treatment of alexia without agraphia is accomplished by rehabilitation by multiple oral re-reading techniques [10]. It is important to suspect this condition based on the nature of the presentation and avoid any delays in beginning an appropriate treatment to prevent further episodes of the stroke while ensuring patients about its nonprogressive course.

## Conclusions

Alexia without agraphia, also known as pure alexia, is a condition when a patient can not read what he/she writes due to a lesion in the visual word form area. This condition is important as a patient might perceive it as a problem in vision and might consult an ophthalmologist. So, along with a neurologist, an ophthalmologist should also be aware of this neurological deficit. Prompt recognition and treatment aid in quick rehabilitation.
